# Activation of Intracellular Complement in Lungs of Patients With Severe COVID-19 Disease Decreases T-Cell Activity in the Lungs

**DOI:** 10.3389/fimmu.2021.700705

**Published:** 2021-11-24

**Authors:** Mark C. Howell, Ryan Green, Andrew R. McGill, Roukiah M. Kahlil, Rinku Dutta, Shyam S. Mohapatra, Subhra Mohapatra

**Affiliations:** ^1^ Department of Internal Medicine, Morsani College of Medicine, University of South Florida (USF), Tampa, FL, United States; ^2^ James A. Haley Veterans Hospital, Department of Veterans Affairs, Tampa, FL, United States; ^3^ Department of Molecular Medicine, Morsani College of Medicine, University of South Florida (USF), Tampa, FL, United States

**Keywords:** bioinformatics, T-cells, complement, severe COVID-19 disease, lungs

## Abstract

A novel coronavirus, Severe Acute Respiratory Syndrome Coronavirus 2 (SARS-CoV-2), arose late in 2019, with disease pathology ranging from asymptomatic to severe respiratory distress with multi-organ failure requiring mechanical ventilator support. It has been found that SARS-CoV-2 infection drives intracellular complement activation in lung cells that tracks with disease severity. However, the cellular and molecular mechanisms responsible remain unclear. To shed light on the potential mechanisms, we examined publicly available RNA-Sequencing data using CIBERSORTx and conducted a Ingenuity Pathway Analysis to address this knowledge gap. In complement to these findings, we used bioinformatics tools to analyze publicly available RNA sequencing data and found that upregulation of complement may be leading to a downregulation of T-cell activity in lungs of severe COVID-19 patients. Thus, targeting treatments aimed at the modulation of classical complement and T-cell activity may help alleviate the proinflammatory effects of COVID-19, reduce lung pathology, and increase the survival of COVID-19 patients.

**Graphical Abstract:**
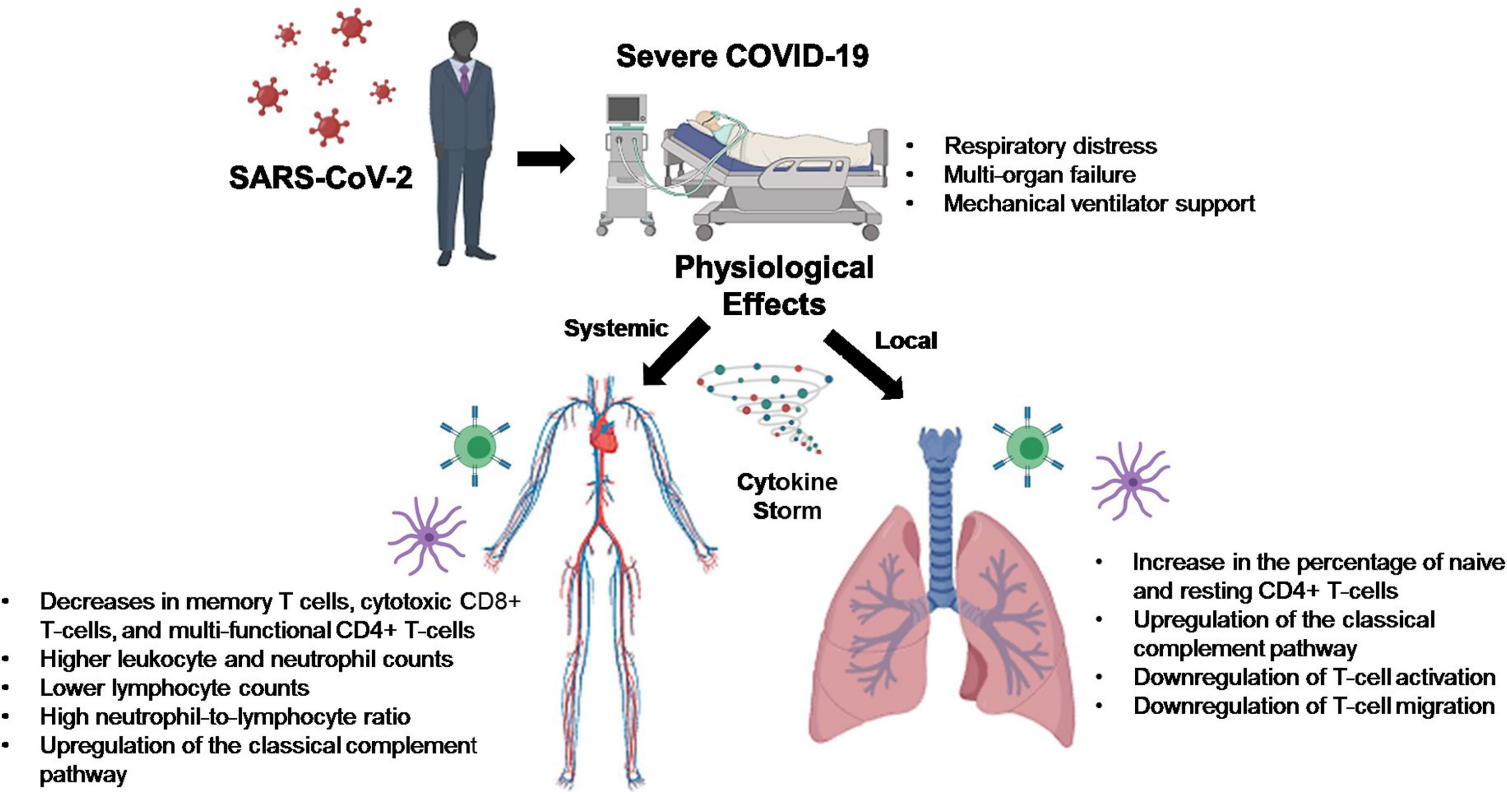


## Introduction

A novel coronavirus, Severe Acute Respiratory Syndrome Coronavirus 2 (SARS-CoV-2), arose late in 2019 in Wuhan, China, which led to a global pandemic resulting in thus far over 239 million confirmed cases and almost 5 million deaths attributed to this virus, as of October 2021, according to the World Health Organization (WHO). Disease pathology ranges from asymptomatic to minimal symptoms, mildly symptomatic with cough and shortness of breath, to severe respiratory distress with multi-organ failure requiring mechanical ventilator support. COVID-19–associated morbidity and mortality have been attributed to a hyperinflammatory cytokine storm caused by strong immune activation (1). COVID-19 has also been shown to suppress host functional adaptive and innate immunity resulting in unrestrained viral dissemination and organ damage (1).

Critical illness in COVID-19 can be traced to several genes found to function in at least two distinct biological pathways: 1) innate antiviral defenses, which are vital early in disease (IFNAR2 and OAS), and 2) host-driven inflammatory lung injury, which is a crucial mediator of life-threatening COVID-19 (DPP9, TYK2, and CCR2) (2). The 3p21.31 gene cluster includes the genes SLC6A20, LZTFL1, CCR9, FYCO1, CXCR6, and XCR1 and has also been identified as a genetic susceptibility locus in COVID-19 patients with respiratory failure (3). However, the cellular mechanisms responsible for the observed increased risk of the severe disease remain unclear and may represent a therapeutic opportunity. To complement these findings, we used bioinformatics tools to analyze publicly available RNA sequencing data from lung biopsies of severely ill COVID-19 patients.

## Materials and Methods

### RNASEQ Dataset Acquisition

With the intent to investigate SARS-CoV-2 infection and immuno-pathology, publicly available RNA-Sequencing datasets were downloaded from the National Center for Biotechnology Information (NCBI) Gene Expression Omnibus (GEO) database (4). These datasets contained RNA-Sequencing data acquired from recently deceased SARS-CoV-2 patients lung biopsies and lung biopsies from recently deceased control patients. Count tables measuring gene expression were downloaded. Data was obtained from three separate studies (EXP1, EXP2, and Live Control). The gene expression data from these RNA-Sequencing experiments was used for both calculation of differential gene expression and CIBERSORTx analysis.

- Deceased SARS-CoV-2 Patients [Experiment # 1 **EXP1**- National Center for Biotechnology Information (NCBI) Gene Expression Omnibus (GEO) (4); GEO Accession GSE147507; Samples- GSM4462413, GSM4462414, GSM4462415, GSM4462416 (5)].
- Deceased SARS-CoV-2 Patients (Experiment #2 **EXP2-** NCBI GEO accession GSE150316; Samples- GSM4546576, GSM4546577, GSM4546578, GSM4546581, GSM4546582, GSM4546584, GSM4546586, GSM4546588, GSM4546589, GSM4546592, GSM4546596, GSM4546596, GSM4546597, GSM4546598, GSM4546599, GSM4546601, GSM4546608, GSM4546609, GSM4546610, GSM4546611, GSM4546612).
- Live Control Patients [NCBI GEO accession GSE83717; Samples; GSM2214000, GSM2214001, GSM2214002, GSM2214003, GSM2214004 (6)].

### Differential Gene Expression Analysis

Differential gene expression was then calculated from the count tables using DESeq2 (7). Gene fold changes representing SARS-CoV-2 infected patients (Either EXP1 or EXP2) against control, meaning the values corresponding to up or down regulations of genes in SARS-CoV-2 samples, were considered significantly differentially expressed if the corrected p-value was <.05.

### CIBERSORT

To estimate the relative percentage of different immune cell types in SARS-CoV-2 patients (Either EXP1 or EXP2) compared to control, we used CIBERSORTx. CIBERSORTx is an analytical tool that provides an estimation of the abundances of member cell types in a mixed cell population using gene expression data (8). Mapped read counts for each sample (Either EXP1 or EXP2) were input into CIBERSORT along with the standard “LM22” gene signature file. CIBERSORT output is displayed as fractional proportions of each immune cell sub-population.

### Pathway Analysis

Furthermore, Ingenuity Pathway Analysis (IPA) (Qiagen) was then used for analysis and interpretation of the acquired differential expressed gene lists from RNA sequencing (9). A detailed description of this software is available at *ingenuity.com*. First, we produced a list containing genes that were significantly changed in at least one dataset and were also commonly up or down regulated in both datasets. Then My Pathway/Path Designer tools were then used in IPA to plot all known interactions between the gene list and top cellular functions of interest.

## Results

**Figure 1:**
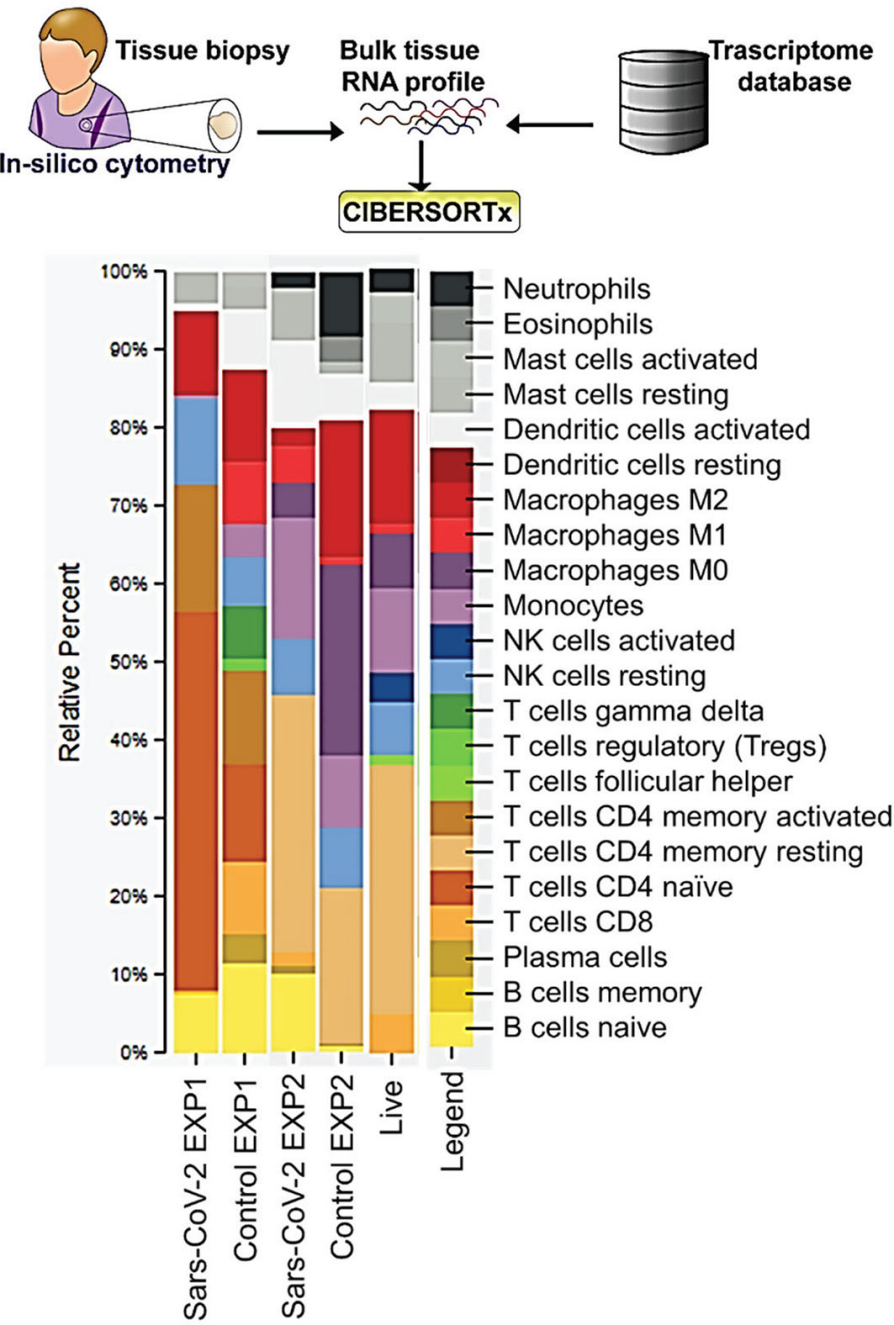
CIBERSORT Analysis- Cell Fractions: This module enumerates the proportions of distinct cell subpopulations in bulk tissue expression profiles.

We used bioinformatics approaches to explore the lung immunity in severely ill COVID-19 patients. CIBERSORTx allows us to estimate relative abundances of immune cells in a sample from gene expression data. The CIBERSORTx results from EXP1 data show a 4-fold increase in naive CD4 T-cells and about a 2-fold increase in resting natural killer (NK) cells in the lungs of postmortem SARS-CoV-2 patients compared to control (**Figure 1**). We also observed decreases in CD8 T-cells, Tregs, gamma/delta T-cells, monocytes, and a 4-fold reduction in activated dendritic cells (**Figure 1**). In EXP2 we observed almost a 2-fold increase in memory resting CD4 T-cells, monocytes, and activated dendritic cells, about a 3-fold increase in mast cells, a 4-fold increase in M1 macrophages, and a 10-fold increase in naive B-cells (**Figure 1**). We also observed decreases in eosinophils, M0 macrophages (5-fold), M2 macrophages (5-fold), and neutrophils (3-fold) (**Figure 1**). The CD4 T-cells in the lungs are mostly identified as naive or resting CD4 T-cells, which may be functionally exhausted at this end stage of the disease (**Figure 1**). Due to the small number of samples in each experiment, one would expect differences in the obtained results. To overcome this limitation, we chose to focus on similarities found in the results, as differences found in the CIBERSORTx results could be attributed to statistical noise and/or patient variation.

Pathway analysis of differentially expressed genes in severe COVID-19 patients showed an upregulation of genes involved in the classical complement pathway, as well as a downregulation of genes involved in T-cell activation and T-cell migration (**Table 1**). Analysis of COVID-19 patients has shown that proteins involved in the complement system are significantly upregulated in the sera of the severe COVID-19 patients (10). The gene C1S, associates with two other complement components C1r and C1q in order to yield the C1 complex, which then triggers the subsequent steps of the classical pathway of complement activation, and is found to be upregulated in the lungs of severe COVID-19 patients in our IPA analysis (11). Severe COVID-19 patients also show an increase in IgG1 production (12). Our IPA analysis found that the IgG component genes, IGHG3, IGHG4, and IGHV3-30 are upregulated in the lungs of severe COVID-19 patients (**Table 1**). These proteins are known to specify effector functions, such as activation of complement or binding to Fc receptors (13).

**Table 1:** Differentially expressed genes in severe COVID-19 patients.

Phenotype	Regulation	Genes
**Activation of T-Lymphocytes**	**Up**	ADA, CARD11, CD38, CD48, CORO1A, DDX58, DEF6, DOCK2, GZMA, ICOS, IL7, PARP1, PIK3CG, PRL, SEMA4A, TNFSF14
	**Down**	AHNAK, AXL, CD44, CD59, CD83, DIABLO, HDAC6, HYOU1, IL24, MAPK14, MAPK8, MAPK9, MR1, NBR1, PAG1, PBX1, PELI1, PRKAA1, PTGER4, RBPJ, RHOB, RUNX1, TICAM1, TLN1, TNFSF9, TYRO3, VAV2, YAP1
**T-Lymphocyte Markers**	**Up**	ADA, CARD11, CCL19, CD38, CD3D, CD48, CHI3L1, CIITA, CORO1A, DDX58, DEF6, DOCK2, ICOS, IL7, MYBL2, PARP1, PIK3CG, POU2AF1, PRKCB, PRL, PSMB10, RARG, TCIRG1
	**Down**	ABL1, AKT3, CD44, CD83, CMTM6, CTSS, DIABLO, DIAPH1, DICER1, ELAVL1, FGFR2, FLT3, HRAS, IL4R, KAT6A, MAML1, MAPK14, MAPK8, MAPK9, MR1, NUP98, PAG1, PBX1, PIP4K2C, PPIA, PRKCH, RBPJ, RIPK1, RUNX1, S1PR2, SERPINB6, SHC1, SOCS3, STK4, TICAM1, TLN1, TNIP1, VAV2, WLS, XRCC6, ZFP36
**Leukocyte Migration**	**Up**	ADA, C1q, CCL19, CD38, CD48, CHI3L1, CIITA, CORO1A, DDX58, DEF6, DOCK2, ICOS, IGHV3-30, IGKV1-12, IGKV4-1, IGLV3-25, IGLV3-27, IL7, LRP6, PARP1, PIK3CG, POU2AF1, PPARA, PRKCB, SEMA4A, STAT1, TCIRG1, TNFSF14
	**Down**	ABL1, AXL, CD44, CTSS, DIAPH1, DICER1, ELAVL1, HDAC6, HRAS, IL24, IL4R, MAML1, MAPK14, MAPK8, MAPK9, MR1, PELI1, PIP4K2C, PPIA, PRKAA1, PTGER4, RHOB, RIPK1, RUNX1, S1PR2, SHC1, SOCS3, STK4, TICAM1, TLN1, TNFSF9, TNIP1, VAV2, WLS, YAP1
**Classical Complement Pathway**	**Up**	C1S, IGHG3, IGHG4, IGHV3-30, IGKV1-12, IGKV4-1, IGLV3-25, IGLV3-27
	**Down**	N/A

Selected cellular functions were chosen from an IPA generated list of significantly altered cellular functions. This was calculated using a list of significantly differential expressed genes common to both datasets. A gene was counted as upregulated if the average expression calculated from both datasets was >1 and downregulated if the average expression was <1.

**Figure 2:**
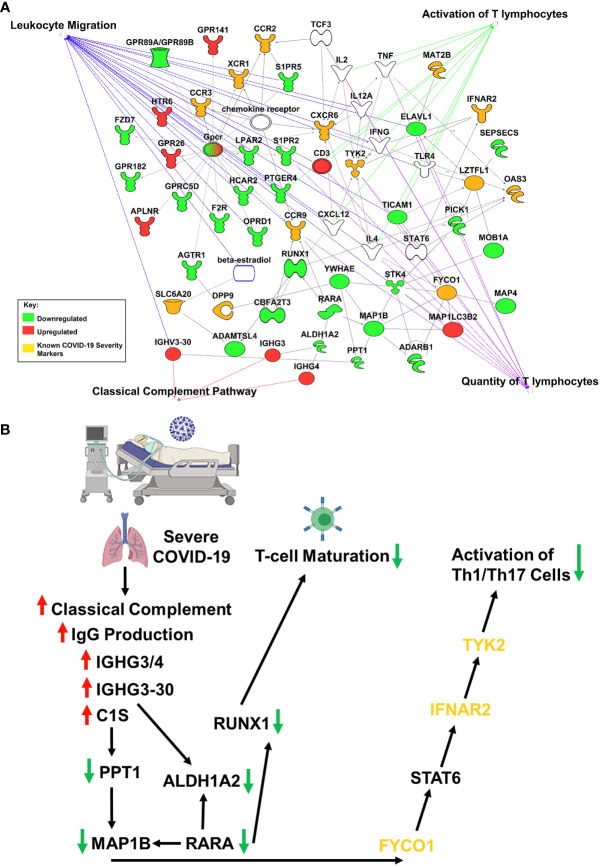
IPA Network Analysis. **(A)** IPA generated network map showing known connections among the common genes found in both RNASeq datasets and the previously identified severe COVID-19 genetic susceptibility markers (SLC6A20, LZTFL1, CCR9, FYCO1, CXCR6, XCR1, DPP9, MAT2B, OAS3, TYK2, CCR2, CCR3, and INFAR2) with known pathways in IPA for cellular functions of interest. Genes are colored based on if the average gene expression between the two experiments showed upregulation (red) or downregulation (green). SLC6A20, LZTFL1, CCR9, FYCO1, CXCR6, XCR1, DPP9, MAT2B, OAS3, TYK2, CCR2, CCR3, and INFAR2 are all labeled in yellow. **(B)** COVID-19 causes upregulation of IgG production leading to activation of classical complement through C1S, which produces a downregulation of RUNX1 and TYK2 signaling, that blocks T-cell maturation and mutes Th1/Th17 immune response, respectively.

Studies have revealed new genetic associations with critical illness in COVID-19 patients (2, 3). Further examination of the interactions between our findings and the previously identified severe COVID-19 genetic susceptibility markers revealed that some of these genes are likely to influence the migration, as well as the antiviral and proinflammatory activity of T-cells (**Figure 2A**). Interestingly, OAS3 was identified as a genetic susceptibility marker in severe COVID-19 disease and is found to be differentially expressed in severe COVID-19 patients in our analysis. OAS3 can also act as a negative regulator of chemokines’ expression and interferon responsive genes in human macrophages (14). We found this gene to be down-regulated in severe COVID-19 cases, which would help explain the highly inflammatory state seen in these patients.

Our results suggest that COVID-19 is causing upregulation of IgG production leading to activation of classical complement through C1S, which produces a downregulation of RUNX1 and TYK2 signaling, that blocks T-cell maturation and mutes Th1/Th17 immune response, respectively (**Figure 2B**). It is important to note that the data obtained in our studies is informatics-based, so further mechanistic studies will need to be performed to confirm the associations found here.

## Discussion

Our results are in contrast with the data obtained from peripheral blood samples of COVID-19 patients, as this shows decreases in total lymphocytes, CD4+ T-cell, CD8+ T-cell, B-cell, and NK cell counts (15). One possible explanation for this observed peripheral lymphopenia could be the sequestration of lymphocytes in the lungs (15). The initial innate immune response to respiratory infections facilitates the recruitment of various immune cell subsets that accumulate in the airways in response to combinations of distinct trafficking signals expressed by airway and alveolar blood vessel endothelial cells (16). It could be that blood vessels in the lung are the first to be affected by SARS-CoV-2-mediated pathology and this may result in a vicious cycle of vessel damage, inflammation, and additional leukocyte recruitment (16). In EXP2 we observed increases of dendritic cells, monocytes, and macrophages. These cells are known to form the mononuclear phagocyte (MNP) system, which sense and phagocytose pathogens, mediate leukocyte recruitment, initiate and shape immune responses and regulate inflammation (17). It has been suggested that during COVID-19 these cells release proinflammatory cytokines resulting in erratic infiltration of pro-inflammatory effector cells, which in turn exacerbates tissue damage (17).

COVID-19 patients with the severe disease show higher leukocyte and neutrophil counts with lower lymphocyte counts and a high neutrophil-to-lymphocyte ratio, as well as lower percentages of monocytes, eosinophils, and basophils (18). In COVID-19 patients, T cell counts are reduced significantly, and the surviving T cells appear functionally exhausted, possibly through SARS-CoV-2-induced NKG2A expression, which may result in disease progression (19). This helps explain the fact that we saw an increase in the percentage of CD4 T-cells in the lungs of SARS-CoV-2 infected patients, as these CD4 T-cells were mainly naïve or resting.

It has been shown that the uncontrolled production of cytokines in critically ill patients with COVID-19 pneumonia leads to cytokine storm syndrome, which is centrally involved in the exacerbation of symptoms and disease development (20). Emerging evidence suggests that the complement system plays a key role in this inflammatory reaction (21, 22). Complement is activated by three main pathways (lectin, alternative, and classical), leading to the production of C3 and C5, which are then cleaved to release C3a and C5a (23). Complement activation is known to suppress viral invasion, however, induction of the cytokine storm by SARS-CoV-2 infection can lead to vascular leakage, activation of complement, diffuse intravascular coagulation and death (20–22). ACE2 expression on vascular endothelium allows for infection by SARS-CoV-2, which induces injury, activates complement, and sets up an inflammation feedback loop (22).

SARS-CoV-2 infection caused activation of complement proteins, including C5b-9, C4d, MASP-2, C1R, C1S, CFB, and complement C3, to be produced locally in the lungs, where they were then processed to activate fragments that caused an upregulation of inflammatory signaling (23, 24). Complement has also been shown to be actively involved in the negative control of T-cell effector immune responses and homeostasis (25). Our IPA analysis predicts that complement activation by SARS-COV-2 infection could be blocking T-cell maturation through a downregulation of RUNX1 signaling. RUNX1 is a transcription factor that is required for T-cell maturation and homeostasis, as it has been shown that RUNX1 deficient T cells bind IgM, C1q, C4 and C3 and are eliminated by complement (26). IPA analysis also predicts that a downregulation of TYK2 signaling, caused by complement activation, could be inhibiting the activation of a Th1/Th17 cellular immune response. TYK2 has been shown to play a role in both the IL-12/Th1 and IL-23/Th17 signaling cascades (27). However, the consequences of this local complement activation within the lung and the correlation with COVID-19 severity are not fully understood. Thus, targeting complement may help alleviate the proinflammatory effects of COVID-19, reduce lung pathology, and thus increase the survival of COVID-19 patients (28). However, further studies will be needed to determine the exact role that SARS-CoV-2 induced complement activation has on the negative regulation of T-cells.

## Conclusion

In summary, severe COVID-19 patients’ lungs exhibit an increase in naive and resting CD4 T-cells. Results suggest that an increase in IgG proteins and complement pathway activation may be leading to the downregulation of CD4 T-cell activation in severe COVID-19 patients (**Figure 2B**). Furthermore, genes found in COVID-19 severity could be influencing the antiviral and proinflammatory activity of these T-cells. Overall, these results suggest that treatments aimed at the modulation of classical complement and T-cell activity may help alleviate the proinflammatory effects of COVID-19, reduce lung pathology, and increase the survival of COVID-19 patients. Further study on the pharmaceutical modulation of these pathways may help reduce the severity of disease in future COVID-19 patients.

## Data Availability Statement

The datasets presented in this study can be found in online repositories. The names of the repository/repositories and accession number(s) can be found below:

https://www.ncbi.nlm.nih.gov/geo/, GSE147507

https://www.ncbi.nlm.nih.gov/geo/, GSE150316

https://www.ncbi.nlm.nih.gov/geo/, GSE83717.

## Author Contributions

Literature gathering and analysis: MH, RG, AM, RK, RD, SM, and SSM. Ingenuity Pathway Analysis: MH, RG. Wrote the manuscript: MH, RG, AM, RK, RD, SM, and SSM. Figure illustration: MH, RG, RK, SM, and SSM. The authors read and approved the final manuscript.

## Funding

This work is supported by Veterans Affairs Merit Review grant (BX005490) to SM and Research Career Scientist Awards to SM (IK6BX004212) and SSM (IK6 BX003778).

## Author Disclaimer

Though this report is based upon work supported, in part, by the Department of Veterans Affairs, Veterans Health Administration, Office of Research and Development, this report’s contents do not represent the views of the Department of Veterans Affairs or the United States Government.

## Conflict of Interest

The authors declare that the research was conducted in the absence of any commercial or financial relationships that could be construed as a potential conflict of interest.

## Publisher’s Note

All claims expressed in this article are solely those of the authors and do not necessarily represent those of their affiliated organizations, or those of the publisher, the editors and the reviewers. Any product that may be evaluated in this article, or claim that may be made by its manufacturer, is not guaranteed or endorsed by the publisher.
